# Ascending and Descending of Coronavirus Disease 2019 (COVID-19)

**DOI:** 10.31661/gmj.v9i0.1880

**Published:** 2020-04-10

**Authors:** Hadi Zare Marzouni, Ali Dashtgard, Esmaeil Hamounpeyma

**Affiliations:** ^1^Qaen School of Nursing and Midwifery, Birjand University of Medical Sciences, Birjand, Iran

**Keywords:** Coronavirus, Severe Acute Respiratory Syndrome, Viruses, Nidovirales

## Dear Editor,

Coronaviruses are a large group of viruses ranging from the common cold virus to severe acute respiratory syndrome (SARS). Coronavirus is one of the viruses belonging *Nidovirales* order from *Coronaviridae* family and *Coronavirinae* Subfamily. Coronavirus is subdivided into alpha, beta, gamma, and delta genuses. All of the viruses in this order are enveloped and have an unusually large non-segmented positive-sense RNA virus genome, and a unique strategy of replication [1,2]. Coronaviruses could create a manifold of diseases in birds and mammals such as enteritis in cows, pigs, and upper respiratory disease chickens [2]. Sometimes, some type of coronaviruses that infected birds and mammals can develop and cause disease in humans and become a new human coronavirus. Studies show that viruses commonly present in either alpha (HCoV-229E and HCoV-NL63) or beta (HCoV-OC43 and HCoV-HKU1) species cause disease in humans, that the most important disease is potentially lethal human respiratory infections [1,3]. The Middle East respiratory syndrome coronavirus (MERS-CoV), SARS-CoV, and SARS-CoV-2 are three types of new human coronavirus that have been caused by the development of coronavirus in animals [1,3]. The SARS-CoV-2 or nCoV-2019 is a novel coronavirus that causes COVID-19, that epidemic started from Wuhan, China in late 2019 and spread to other countries and About 1 million people in at least 200 countries have been infected, and about 57000 have died at the time of writing (04 April 2020) [4]. World statistics of COVID-19 show that the incidence and mortality rate of nCoV-2019 is much higher than SARS-CoV and Influenza A (H1N1) virus [5]. COVID-19 causes infections in the nose, sinuses, and gliomas and is associated with fibrosis in the lungs. Studies showed that the human angiotensin-converting enzyme 2 (ACE-2) has a very high-affinity binding to nCoV-2019 [6,7]. This receptor is abundant on the surface of the heart and lung cells. For this reason, the nCoV-2019 is the main attack on these tissues. Besides, this receptor is expressed on the surface of adipose tissue, kidney and cancerous cell [6]. Therefore, people with diabetes, heart and lung disease, immune system disorder, pregnancy, cancer, and obese individuals are at risk for COVID-19 [5,6]. Also, due to the high transmission rate, this disease is a serious public health threat worldwide [8]. One of the factors that can increase the prevalence of COVID-19 is the high incubation period of nCoV-2019, which ranges from 2 to 14 days. During this period, the infected person carries the virus without any symptoms, and could transmit it to other persons [2,9]. The remarkable note is that viral infections usually spread very quickly at the onset, and subside after a while. It seems one of the major causes of the infection subsides is the adaptation of the immune system to the virus and the development of appropriate immune responses to the virus [3]. In addition, the serial passages and replication of the virus in the host body and its transmission to subsequent hosts have caused mutations in the virus genome that can alter its pathogenicity. However, the roles of specific mutations and their effect on virus pathogenicity or adaptation are mainly unknown and require more study and examination [10]. Although the evidence indicated that the prevalence of COVID-19 is increasing; however, it can be hoped to reduce its incidence in the near future. It seems that considering the health of the mucosal cells in the respiratory system, the consumption of vitamin D3, and increase in the level of the immune system could be very effective in preventing and improving the COVID-19. Moreover, the hygiene compliance, avoid entering densely populated areas and pay attention to health advice, can be very effective in controlling and reducing the incidence of COVID-19 at this critical period of the epidemic [3,5,2].

## Acknowledgment

 A series of studies on COVID-19 Emerging Disease by the authors of this study were conducted at the Laboratory of Qaen School of Nursing and Midwifery, Birjand University of Medical Sciences, Birjand, Iran. The authors of this article are sincerely appreciated by all who have helped us, especially Dr. Mohammad Reza Arefifar.

## Conﬂict of Interest

 The author(s) indicated no conﬂicts of interest.
